# Can fearlessness come in a tiny package?

**DOI:** 10.7554/eLife.24575

**Published:** 2017-02-06

**Authors:** Bryan W Luikart

**Affiliations:** Geisel School of Medicine, Dartmouth College, Hanover, United StatesBryan.W.Luikart@dartmouth.edu

**Keywords:** learning, memory, synaptic plasticity, miRNA, hippocampus, negative feed back loop, Rat

## Abstract

A molecule called microRNA-153 helps to prevent rats associating new environments with fear.

**Related research article** Mathew RS, Tatarakis A, Rudenko A, Johnson-Venkatesh EM, Yang YJ, Murphy EA, Todd TP, Schepers ST, Siuti N, Martorell AJ, Falls WA, Hammack SE, Walsh CA, Tsai LH, Umemori H, Bouton ME, Moazed D. 2016. A microRNA negative feedback loop downregulates vesicle transport and inhibits fear memory. *eLife* **5**:e22467. doi: 10.7554/eLife.22467

Contextual fear conditioning is a process that occurs when a painful or frightening stimulus happens within a specific context, and it can cause an individual to fear the context even when the stimulus is removed. In humans, it is thought that contextual fear conditioning can contribute to anxiety and post-traumatic stress disorder, so understanding how the brain forms “associative memories” that link a context to a traumatic event has been the subject of research for many years (Maren et al., 2013).

In rodents, we can study the neurobiological mechanisms responsible for the formation of such memories by pairing a painful stimulus with a new environment. If a rodent experiences an electrical shock after being placed in a new cage, it will associate that cage with the electrical shock. Thus, when the rodent is placed in the cage after this association has been established, it will become “frozen with fear” even if no shock is delivered.

Two regions of the brain – the amygdala and the hippocampus – have major roles in the formation of fear-associated memories. The experience of fear increases neuronal activity in several regions of the amygdala, and learning about a new environment increases activity in the hippocampus (Phillips and LeDoux, 1992). Stable associative memories are formed by changing the strength of the connections between neurons – called synapses – in these two regions.

To communicate across synapses, the presynaptic neuron releases neurotransmitters from membrane-enclosed compartments called vesicles in a process called exocytosis. The neurotransmitter molecules then travel across the synapse and bind to receptors on the surface of the postsynaptic neuron. The strength of the synapse can be changed by altering the ability of the presynaptic neuron to release neurotransmitters, or by altering the availability of the receptors on the postsynaptic neuron (Kessels and Malinow, 2009; Nicoll and Schmitz, 2005). Now, in eLife, Danesh Moazed of Harvard Medical School and colleagues – including Rebecca Mathew and Antonis Tatarakis as joint first authors – report that the synapses responsible for the formation of fear-associated memories are kept in check by a tiny molecule called microRNA-153 (Mathew et al., 2016).

MicroRNAs are short RNA molecules (Lee et al., 1993; Wightman et al., 1993) that interfere with the ability of messenger RNA molecules to encode proteins (Selbach et al., 2008). Mathew et al. – who are based at Harvard and a number of other institutes in the United States – identified a set of 21 microRNAs whose production is increased by fear conditioning in rats. In particular, they found that learning to associate fear with a new environment caused the expression of microRNA-153 to increase by a factor of approximately four in a part of the hippocampus called the dentate gyrus.

To determine whether microRNA-153 has a role in the formation of fear-associated memories Mathew et al. reduced its production in the hippocampus and performed fear conditioning experiments. They found that rats that were deficient in microRNA-153 froze more often in the cage where they had experienced an electrical shock. Thus, it appears that microRNA-153 decreases the formation of fear-associated memories.

To determine how microRNA-153 inhibits the formation of fear memories, Mathew et al. analyzed all of the genes that they had predicted would be regulated by fear-induced microRNAs. This sample included a large proportion of the genes involved in vesicle exocytosis, and microRNA-153 targeted a large number of these genes. Further investigation revealed that fear conditioning reduced the expression of the exocytosis-related genes, and microRNA-153 knockdown increased their expression.

Genes regulated by microRNA-153 (such as *Snap25* and *Pclo*) control synaptic strength by regulating both presynaptic vesicle exocytosis and postsynaptic receptor trafficking (Jurado et al., 2013; Südhof, 2013). By demonstrating in vitro that manipulating the expression of microRNA-153 can also regulate these processes, Mathew et al. conclude that microRNA-153 counteracts the formation of associative memories during fear conditioning by decreasing the strength of synapses.

The results also lead to a number of new questions. Does microRNA-153 regulate the activity of the hippocampus more generally? And is microRNA-153 expression regulated in other brain regions, such as the amygdala, to modulate other aspects of fear?

It is also important to note that Mathew et al. found a total of 21 microRNAs whose production increased as a result of fear conditioning. Based on their sequence, these microRNAs are predicted to target genes involved in a number of processes: vesicle fusion, neuronal development, long-term potentiation, neurotransmission and synaptogenic adhesion. Thus, figuring out how these microRNAs influence memory formation is likely to involve a number of mechanisms that were not investigated by Mathew et al. Finally, as we gain insight into the roles that microRNAs play, it may be possible to leverage the properties of these tiny molecules to develop new treatments for anxiety and other disorders.
